# The Journal—New Series—Volume V

**Published:** 1888-03

**Authors:** 


### THE JOURNAL—NEW SERIES—VOLUME V.

With this issue THE JOURNAL enters upon the thirtieth year of its existence and the fifth of the new series. With a full desire to place it among the leading medical journals of this country, its successive administrations have worked with untiring zeal, have borne with commendable patience its trials and troubles, as step by step it marched to the front rank of medical journalism. But it has been left the present management to enjoy the full fruition of the labors that have been spent, to reap the well-earned rewards that have been bestowed by an appreciative public. Intelligently managed, it now stands on a foundation tha cannot be shaken. Its contributors are among the most reliable and distinguished in the country, and nothing will be left undone in the future to maintain it in the position it has taken and to make its pages such that, not only the most learned and scientific can understand, but the most practical and hard-worked physician may appreciate and enjoy. No pains will be spared, no labor left undone, to make each succeeding number more practical and interesting than its predecessor, so that THE JOURNAL will become an indispensable part of the medical library of the general practitioner. It takes scientific skill, tact, industry and money to successfully conduct a medical journal ; it takes labor that ceases not, as the weeks merge into months and the months into years, and we hope we are not asking too much of our readers for their hearty and earnest coöperation in all our efforts to increase the usefulness and reputation of the ATLANTA MEDICAL AND SURGICAL JOURNAL.

